# Cork-Derived Carbon Sheets for High-Performance Na-Ion Capacitors

**DOI:** 10.1021/acsaem.3c01212

**Published:** 2023-07-17

**Authors:** María Dolores Casal, Noel Díez, Sara Payá, Marta Sevilla

**Affiliations:** Instituto de Ciencia y Tecnología del Carbono (INCAR), CSIC, Francisco Pintado Fe 26, 33011 Oviedo, Spain

**Keywords:** cork, carbon material, carbon sheets, S-doping, ball milling, sodium-ion capacitor

## Abstract

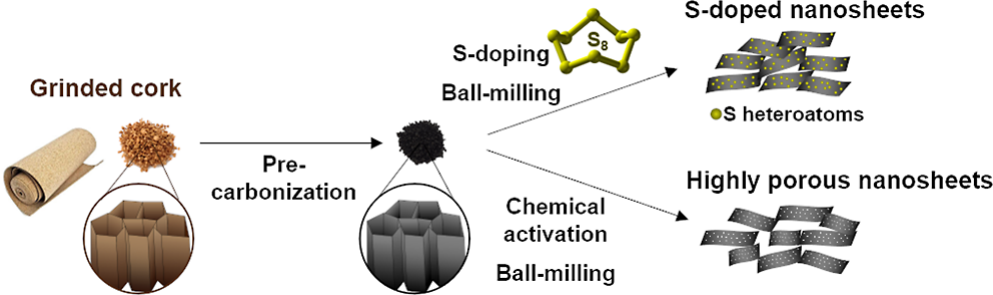

S-doped carbon sheets have been easily prepared by deconstructing the 3D cellular structure of a fully sustainable and renewable biomass material such as cork through a mild ball-milling process. S-doping of the material (>14 wt % S) has been achieved by using sulfur as an earth-abundant, cost-effective, and environmentally benign S-dopant. Such synthesized materials provide large Na storage capacities in the range of 300–550 mAh g^–1^ at 0.1 A g^–1^ and can handle large current densities of 10 A g^–1^, providing 55–140 mAh g^–1^. Their increased packing density compared to the 3D pristine structure allows them to also provide good volumetric capacities in the range of 285–522 mAh cm^–3^ at 0.1 A g^–1^ and 53–133 mAh cm^–3^ at 10 A g^–1^. In addition, highly porous carbon sheets (*S*_BET_ > 2700 m^2^ g^–1^) have been produced from the same carbon precursor by rationally designing the chemical activation approach. These materials are able to provide good anion storage capacities/capacitances of up to 100–114 mAh g^–1^/163–196 F g^–1^. A sodium-ion capacitor assembled with the optimized S-doped carbon sheets and the highly porous carbon sheets with mass matching ratios provided the best energy/power characteristics (90 Wh kg^–1^ at 29 kW kg^–1^) in combination with robust cycling stability over 10,000 cycles, with a capacity fade of only 0.0018% per cycle.

## 1 Introduction

We live in an era of constant technological innovations which steadily demand more powerful energy storage and conversion solutions. In particular, the increasing portabilization of electronic devices and the ineludible electrification of the transport sector necessary to meet the European Union goals of decarbonization and energy security and urge to find energy storage technologies combining high energy, high power, long cycle life, and safety, in addition to low environmental impact. Within this context, hybrid ion capacitors have gathered increasing attention since they combine the high energy density and low self-discharge of batteries with the high power density and long cycle life characteristic of supercapacitors. This combination of characteristics arises from the use in the same device of a battery-type material generally at the negative electrode—where the storage of cations in the bulk takes place by insertion, conversion, alloying, or pseudocapacitive mechanisms—and a capacitor-type material at the positive electrode—which stores the anions by means of surface adsorption/desorption. As a result, these devices are a main candidate for regenerative braking and power assistance in automotive applications, backup for emergency power regeneration, power leveling for stabilizing energy grids, or to power the Internet of Things (IoT).

Li-ion capacitors (LICs) were the first to appear and are already commercialized since 2008.^1^ However, the limited availability and geopolitical constraints of lithium resources with the associated price increase and extraction-derived environmental issues have prompted the interest in Na-ion capacitors (NICs), similar to their battery counterparts. Thus, sodium resources are characterized by their abundance and widespread geographical distribution, which, in combination with the use of aluminum instead of copper as a negative electrode current collector, promises to substantially decrease the price of the devices. Besides, sodium-based hybrid systems have been reported to be able to match the performance of their lithium-based counterparts.^2^ The first reports on NICs date back to 2012 and integrated a commercial activated carbon as the positive electrode and, as the negative electrode, V_2_O_5_/CNT nanowire composites,^3^ sodium titanate,^4^ or a commercial hard carbon.^5^ This last NIC is what has been labeled as a dual-carbon NIC. Since both electrodes are made up of carbon materials, these devices benefit from the cost-effectiveness, sustainability, recyclability, and wide tunability of the physicochemical properties of carbon materials.^6^ For the positive electrode, activated carbons or highly porous carbon structures are the material of choice on account of a large accessible surface area capable of electro-adsorbing the electrolyte anions.^7^ In the case of the negative electrode, disordered carbons such as hard carbons are the preferred option by virtue of their enlarged interlayer space (>0.36–0.37 nm) and abundant active sites for Na storage (e.g., structural defects, pores, or heteroatoms), providing a high capacity at low current rates (>300 mA h g^–1^).^8−11^ However, hard carbons suffer from a poor rate performance arising from a bulk diffusion-controlled sodium storage mechanism based on the insertion and de-insertion of the cations. In order to overcome the kinetic mismatch issues between the negative and positive electrodes, which are currently limiting the performance of NICs, two main strategies have been explored: namely, minimization of solid-state diffusion distances by structural/morphology engineering and promotion of pseudocapacitive sodium storage processes by heteroatom doping. Within the first strategy, a variety of carbon nanostructures have been studied, such as carbon spheres^12−14^ or capsules,^15,16^ nanofibers,^17,18^ nanosheets,^2,19^ or 3D porous carbons.^20−22^ On the other hand, heteroatom doping (e.g., N, S, O, or P) has proven to be a powerful tool to boost the pseudocapacitive Na storage performance through defects creation and quick surface redox reactions, besides speeding the diffusion-limited intercalation through the expansion of the interlayer spacing.^17,18,23,24^

However, an important challenge still remaining is the accomplishment of structural engineering and heteroatom doping by simple and sustainable methodologies. This is important in rising technologies whose materials demands—along with their associated environmental footprint—are expected to largely increase. In this regard, the use of biomass as carbon precursor represents a sustainable alternative to the use of fossil fuel-derived precursors, and in the case of biomass residues, it provides a solution to the contamination problem derived from its landfilling or burning. However, for real sustainability, biomass has to be selected so that it does not compete with feeding purposes and it should be renewable, i.e., it should regenerate/grow at a faster rate than it is consumed. In this regard, cork is considered a fully sustainable and renewable biomass material since it is harvested from the periodical regeneration (usually every 9–12 years) of the oak bark and does not involve the cutting down of the tree. Besides, waste cork stoppers and cork dust (which accounts for about 30–40% of total cork production during the manufacturing process^25^) can also be harnessed. Also important is that cork is naturally porous with a unique 3D honeycomb-like structure, which is preserved during carbonization, yielding a carbon material with short diffusional pathways, a feature that is advantageous in energy storage applications. In this regard, Li et al. showed the use of cork-derived carbon as a Na-ion battery anode, providing a high reversible capacity in the range of ∼300–360 mA h g^–1^ at 0.03 A g^–1^ but a limited rate capability owing to the poor kinetics of its dominant pore filling sodium storage mechanism at low potentials. Also, the synthesis temperatures used were high, in the 1200–1600 °C range, which increased the energy consumption and carbon footprint.^26^ In addition, the employment of 3D structures comprising large voids reduces the packing density of the electrode, compromising the energy density of the device. Accordingly, in this work, we address the low-temperature synthesis of S-doped, cork-derived carbon sheets with a high concentration of structural defects for sodium storage, short diffusional pathways, and a suitable packing density achieved by a mild ball-milling step. As the S-dopant, abundant, environmentally benign, and cost-effective elemental sulfur was chosen. In this way, carbon sheets with sulfur contents in the 14–27 wt % range were obtained, with sulfur being covalently bonded to the carbon framework. As a result, these materials showed high Na storage capacities of 300–550 mAh g^–1^ at 0.1 A g^–1^ and 55–140 mAh g^–1^ at 10 A g^–1^. What is more, highly porous carbon sheets with BET specific surface areas above 2700 m^2^ g^–1^ were obtained from the same carbon precursor by rationally designing the chemical activation approach. These materials were analyzed as positive electrode materials, providing ClO_4_^–1^ anion storage capacities/capacitances of up to 100–114 mAh g^–1^/163–196 F g^–1^. NICs were assembled with the S-doped carbon sheets and the porous carbon sheets, and the optimization of their performance in terms of energy/power density, cycling stability, and safety was assessed by evaluating different electrode mass ratios.

## 2 Experimental Section

### 2.1 Synthesis of S-Doped Carbon Materials

First, the corkboard was grinded and sieved to a particle size <1 mm. Second, the cork powder was heat-treated at 400 °C for 1 h (5 °C min^–1^) under nitrogen gas flow. Afterward, it was washed with diluted HCl to remove the intrinsic inorganic matter and then with abundant distilled water and dried at 120 °C for several hours. In a second step, the pre-carbonized cork was thoroughly mixed in a mortar with sulfur (95%, Alfa Aesar) in a wt ratio of 1:3. The mixture was then transferred to a crucible and heat-treated at different temperatures in the 600–750 °C range for 3 h (3 °C min^–1^) under nitrogen gas flow. As a final step, the materials were ball-milled in a Retsch MM200 for 15 min using agate balls (diameter = 6 mm, balls to S-doped carbon wt ratio of 8) and a frequency of 10 s^–1^. For comparison purposes, a sample was prepared following the same procedure but performing the second thermal treatment in the absence of sulfur at a temperature of 700 °C. The materials are labeled as CS-*T*, with *T* being the temperature of the S-doping step, CS-*T*m for the ball-milled materials, and C-700m for the material prepared in the absence of sulfur and subjected as well to a ball-milling process.

### 2.2 Synthesis of Porous Carbon Materials

The positive electrode materials were prepared from cork by means of an activation process using a mixture of hydroxides as chemical activating agents, i.e., NaOH/KOH (wt ratio = 1). In the first step, cork powder was heat-treated at 600 °C for 1 h (5 °C min^–1^) under nitrogen gas flow. In the second step, the pre-carbonized cork was activated using a wt ratio of activating agent to pre-carbonized material of 3. Then, the mixture was transferred into a high alumina crucible and heat-treated at 700–800 °C for 1 h (3 °C min^–1^) under nitrogen gas flow. After washing with 15% HCl to remove the inorganic activation byproducts and abundant distilled water until neutral pH, the porous carbon materials were dried at 120 °C for several hours. Finally, the materials were ball-milled in a Retsch MM200 for 10 min using agate balls (diameter = 6 mm, balls to carbon wt ratio of 8) and a frequency of 10 s^–1^. For the optimized positive electrode material, a post-synthesis thermal treatment at 800 °C for 2 h (heating ramp of 5 °C min^–1^) was further applied.

### 2.3 Physico-Chemical Characterization

Scanning electron microscopy (SEM) images were acquired in a Quanta FEG650 (FEI) instrument, equipped with energy-dispersive X-ray spectroscopy (EDX). The N_2_ adsorption isotherms were measured at −196 °C using a Micromeritics ASAP 2020 sorptometer. The apparent surface area was calculated by applying the BET method, for which an appropriate relative pressure range was selected to ensure a positive line intersect of multipoint BET fitting (*C* > 0). The total pore volume was determined from the amount of nitrogen adsorbed at a relative pressure (*P*/*P*_0_) of 0.95. The pore size distributions (PSDs) were determined by using the quenched solid state density functional theory (QSDFT) method for nitrogen. The packing density and electronic conductivity of the materials were measured by pressing the carbon powders at a pressure of 7.1 MPa with stainless steel rods inside a home-made Teflon cylinder. X-ray diffraction (XRD) patterns were obtained on a Bruker D8 Advance instrument operating at 40 kV and 20 mA using a Cu Kα radiation source. Elemental CHN analysis of the samples was carried out on a LECO TruSpec_micro, whereas the oxygen content was quantified on a LECO TruSpec_micro_o and the sulfur content on a LECO S632. The Raman spectra were recorded on a Horiba (LabRam HR-800) spectrometer. The source of radiation was a laser operating at a wavelength of 514 nm and a power of 25 mW. X-ray photoelectron spectroscopy (XPS) was carried out on a Specs spectrometer using Mg KR (1253.6 eV) radiation from a double anode at 150 W.

### 2.4 Electrochemical Characterization

Slurries were prepared out of the negative and positive electrode materials by mixing them with Super C65 carbon black and carboxymethyl cellulose (CMC) binder (MW = 700,000, Sigma-Aldrich) in a wt % ratio of 80:10:10 and dispersed in water by magnetic stirring overnight. The slurry was then cast onto carbon-coated aluminum foil using the Doctor Blade technique, dried overnight at 105 °C under vacuum, and finally subjected to calendering. Afterward, the coated foil was cut into disks with a diameter of 10 mm (the active carbon mass loading in the electrode is of 1.0–1.2 mg cm^–2^). Half-cell characterization was performed by using CR2032 coin-type cells, which were assembled in an Ar-filled glovebox using a Na metal disk (diameter = 12 mm) as the counter and reference electrode and a Whatman GF/C glass fiber disk as the separator (diameter = 16 mm). The electrolyte consisted of a solution of 1 M NaClO_4_ in a mixture of carbonates (EC/DEC = 1:1 vol). The electrochemical performance of the half-cells was tested at room temperature (24–25 °C) in a computer-controlled potentiostat (Biologic VMP3 multichannel generator). Galvanostatic charge/discharge (GCD) experiments were recorded in the range of 0.01–3.0 V vs Na/Na^+^ in the case of the negative electrode material and in the range of 2.0–4.2 V vs Na/Na^+^ for the positive electrode material using increasing current densities from 0.1 to 10 A g^–1^. Cyclic voltammetry (CV) experiments were recorded within the same potential window used in the GCD experiments at increasing sweep rates in the 0.1 to 1.0 or 5 to 100 mV s^–1^ range for the negative and positive electrode materials, respectively.

Full cells were assembled using a three-electrode configuration (Swagelok T-cell) with a metallic Na reference electrode to record the changes in the potential of the negative and positive electrodes. The current collectors consisted of stainless steel rods, with the separator and electrolyte being the same as those in the half-cells. Before testing, the negative and positive electrodes were pre-conditioned to compensate for the initial sodium losses. Thus, the negative electrode was cycled three times between 0.01 and 3.0 V vs Na/Na^+^ at 0.1 A g^–1^, setting a cut-off potential of 0.05 V vs Na/Na^+^. Meanwhile, the positive electrode material was cycled five times between 2 and 4.2 V vs Na/Na^+^ at 0.1 A g^–1^, setting a cut-off potential of 4.2 V vs Na/Na^+^. Different positive-to-negative active materials weight ratios in the range of 2:1–1:2 were evaluated, keeping constant a total active mass of ≈1.5–2 mg cm^–2^ in the device. The specific capacitance (F g^–1^), specific energy (Wh kg^–1^), and specific power (W kg^–1^) of the device were calculated using the formulae
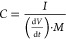
1
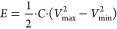
2
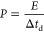
3where *I* is the current, *M* is the total mass of active materials in the positive and negative electrodes, d*V*/d*t* = slope of the discharge curve, Δ*V*_d_ is the operation voltage (*V*_max_ – *IR*_drop_ – *V*_min_), and Δ*t*_d_ is the discharge time.

## 3 Results and Discussion

### 3.1 Structural, Chemical, and Electrochemical Properties of the Negative Electrode Material

**Scheme 1:**
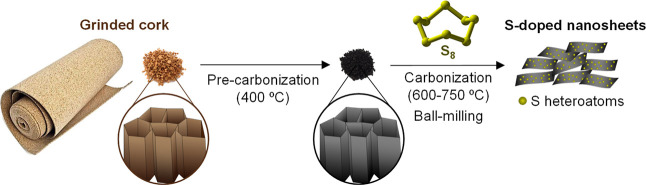
Illustration of the Synthesis Strategy toward S-Doped Carbon Sheets from Cork

The synthesis strategy followed in this work for the production of the S-doped carbon sheets is based on harnessing the honeycomb-like 3D structure of cork made up of interconnected suberized cell walls. As shown in Scheme 1, the cork is first pre-carbonized at a low temperature of 400 °C, followed by its sulfurization with environmentally benign elemental sulfur at temperatures in the 600–750 °C range and a final ball-milling step, which allows deconstruction of the 3D structure into 2D sheets. The yield of the pre-carbonization step is ∼30 wt %, while that of the S-doping step decreases from 90 to 72 wt % with the increase of the synthesis temperature. Consequently, the global yield of the synthesis process falls within the 21–26 wt % range, a value which compares favorably with biomass-derived hard carbons (normally < 20 wt %).^27−31^ This is important from the economic point of view and also from the efficient use of resources viewpoint, especially considering our current excessive consumption rate of resources.

**Figure 1:**
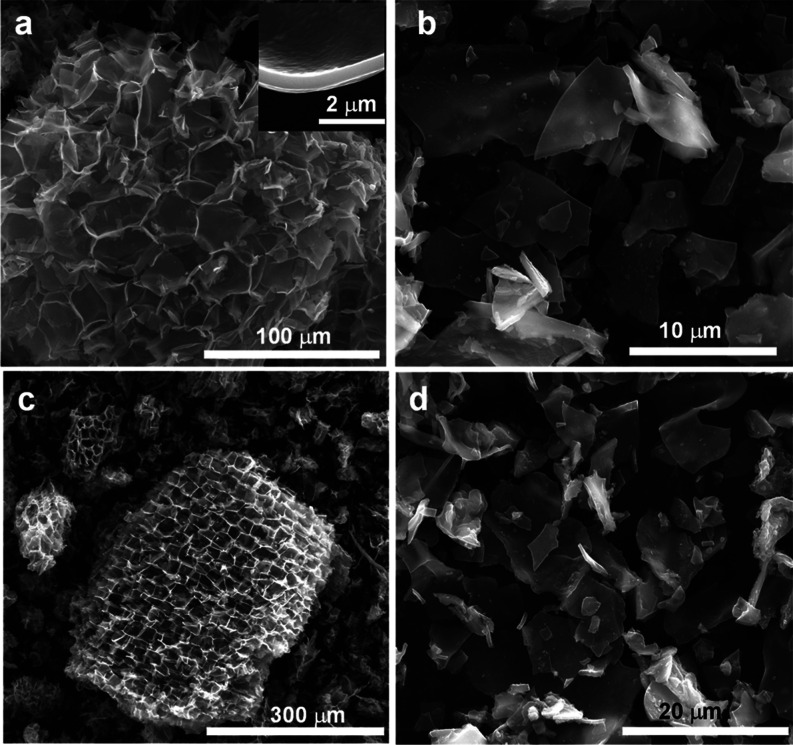
SEM pictures of (a) CS-600, (b) CS-600m, (c) CS-750, and (d) CS-750m.

As mentioned before and revealed by Figure S1a, the cork is characterized by a 3D cellular structure consisting mainly of pentagonal and hexagonal cells combined in a honeycomb-type arrangement. This structure is well preserved after its carbonization at a mild temperature of 400 °C (Figure S1b) and also after its high temperature treatment at 600 and 750 °C in the presence of sulfur (Figure 1a,c). It should be noted, however, that a reduction in the cell wall thickness is registered from 1 to 1.5 μm in pristine cork down to ∼250–450 nm after carbonization at 400–750 °C. Even though this open macroporous structure is advantageous from the diffusion of species standpoint, it endows the material with a low packing density, which is detrimental from an energy density point of view. Accordingly, a mild ball-milling step was implemented in order to deconstruct this 3D structure into the 2D sheets that compose the cell walls, along with some irregular particles with small thickness corresponding to the joints of the cells (Figure 1b,d). The 2D sheets enable a higher packing density of the material, while they retain a suitable morphology that is expected to provide quick access to the active sites in the carbon material. Indeed, the packing density doubles after the ball-milling process, from ∼0.5 g cm^–3^ for the pristine S-doped samples to ∼1 g cm^–3^ (see Table 1).

**Table 1:** Physico-Chemical Properties of the Carbon Sheets

carbon material	global synthesis yield (%)	textural properties		structural properties		chemical properties		packing density (g cm^–3^)^b^	electronic conductivity (S m^–1^)^b^
		*S*_BET_ (m^2^ g^–1^)	*V*_p_ (cm^3^ g^–1^)^a^	*d*_002_ (nm)	*I*_D_/*I*_G_	C (wt %)	S (wt %)		
C-700m	20	81	0.04	0.372	2.72	90.6	0	0.74	9.7
CS-600m	26	430	0.18	0.375	2.54	67.5	26.7	0.95	
CS-650m	25	454	0.18	0.372	2.54	73.2	22.2	1.07	0.2
CS-700m	22	484	0.20	0.373	2.60	75.7	19.8	0.96	2.1
CS-750m	21	534	0.22	0.367	2.71	81.2	14.3	0.95	14.1

a Pore volume determined at (*P*/*P*_0_) = 0.95.

b Determined at 7.1 MPa.

**Figure 2:**
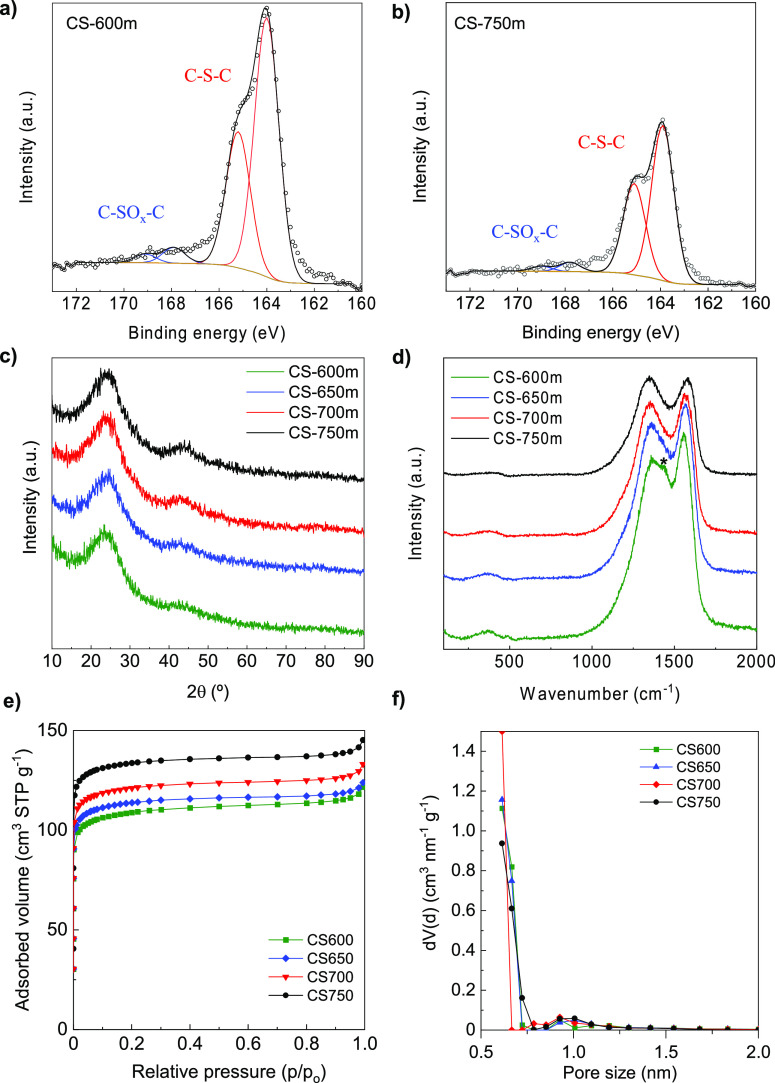
High-resolution XPS S 2p spectra of (a) CS-600m and (b) CS-750m; (c) XRD patterns, (d) first-order Raman spectra, (e) N_2_ adsorption isotherms, and (f) QSDFT PSD of the S-doped carbon sheets.

The effective S-doping of the carbon materials was clearly proved by the elemental chemical analysis of the materials. Thus, regardless of the synthesis temperature, all the materials exhibit large S contents, well beyond 14 wt % and exceeding 20 wt % for the lowest temperatures, i.e., 600–650 °C (Table 1). In addition, all the materials contain 1.4–1.6 wt % N and 2.6–3.7 wt % O. Consequently, the carbon content of these materials lies in the range of 67–81 wt % (compared to 91% for the undoped sample C-700m, which additionally has an O content of 6.5 wt % and a N content of 1.8 wt %). The specific nature of the sulfur functionalities introduced in the carbon framework was assessed by XPS. As revealed by the S 2p spectra in Figure 2a,b for CS-600m and CS-750m, respectively, regardless of the synthesis temperature, sulfur is mainly present as thiophenic-S (C–S_*x*_–C, 164 eV), with a very minor contribution (∼5%) as oxidized sulfur (C–SO_*x*_–C, 167.9 eV). The uniform distribution of these sulfur species along the sheets was confirmed by SEM–EDX, as shown in the elemental mappings in Figure S2. It is worth noting that this sulfur covalently attached to the carbon framework in thiophene-type functionalities has been shown to be an active site for Na storage through its bond cleavage and rearrangement.^32^ Accordingly, a high Na storage capacity arising from redox processes above 0.4 V vs Na/Na^+^ is expected from these materials.

Figure 2c shows the XRD patterns of the sheets synthesized at different temperatures. All the diffractograms are characteristic of highly disordered carbons, i.e., broad and low intensity (002) and (10) diffraction bands are the only distinguishable ones. The average interlayer spacing calculated from the position of the (002) peak by applying the Bragg equation lies in the 0.366–0.375 nm range (Table 1). On the other hand, the undoped carbon sheets, C-700m, show somehow more intense (002) and (10) diffraction bands (Figure S3a), which indicate slightly larger domains of graphitic ordering. However, their average interlayer spacing has a value of 0.372 nm, which is very similar to that of the corresponding S-doped carbon, CS-700m (Table 1). These results suggest that the S-doped carbon sheets have a defective structure in which sulfur is doped at vacancy defect sites on the carbon layer instead of in the interlayer space.^33^ Nevertheless, this dilated interlayer spacing should offer little resistance to Na^+^ diffusion, facilitating its insertion/de-insertion in/out of the material.^34^ The microstructural ordering of the carbon materials was further assessed by Raman spectroscopy. The corresponding first-order Raman spectra are depicted in Figure 2d (and the deconvolution in Figure S3b,c for CS-600m and CS-750m). In addition to the disorder-induced D band at 1350–1360 cm^–1^ and the graphitic order-induced G band at 1570–1580 cm^–1^, all the spectra show bands at ∼370, ∼1180^—^, and ∼1520 cm^–1^, which correspond, respectively, to C–S stretching vibrations, disordered graphitic lattices provided by sp^2^–sp^3^ bands (known as D* band), and amorphous phases (known as D″ band).^35^ Besides, for the materials synthesized at 600 and 650 °C (CS-600m and CS-650m), an additional band attributable to the C=C stretching vibration in thiophene rings develops at ca. 1450 cm^–1^ (especially marked for CS-600m as shown in Figure S3b),^36^ and in the case of CS-600m, a tiny peak appears at ca. 490 cm^–1^ corresponding to S–S stretching vibrations. These results support the fact that S is covalently bonded to carbon atoms and only residual elemental sulfur is present at 600 °C, probably trapped inside the porosity. On the other hand, the value of the integrated intensities (*I*_D_/*I*_G_) increases with the doping temperature (Table 1), indicating a decrease in defects according to stage 2 of the three-stage Raman model proposed by Ferrari and Robertson,^37^ which agrees with the decrease of sulfur heteroatoms and the growth of the aromatic clusters. As a result, the electronic conductivity of the sheets increases with the synthesis temperature (Table 1).

Nitrogen physisorption was used to measure the textural properties of the materials. As can be seen in Figure 2e, all the materials exhibit a type I isotherm, characteristic of microporous materials. The PSDs in Figure 2f show virtually no pores above 2 nm, with most of the porosity being in the narrow micropore range (i.e., <0.8 nm). Table 1 gathers the textural data derived from the analysis of the adsorption isotherms. The materials have BET surface areas in the range of ∼430–530 m^2^ g^–1^, with the value increasing slightly with the synthesis temperature. In contrast, cork carbonized in the absence of sulfur at 700 °C and ball-milled under the same conditions has a BET surface area lower than 100 m^2^ g^–1^, which suggests some porogenic action of sulfur, in addition to its S-dopant role. This effect has also been observed for raw biomass^36^ and polymers.^14^ As will be shown, in spite of the relatively large surface area of the doped materials, the initial Coulombic efficiency (ICE) of the negative electrodes made up of them is considerably high.

**Figure 3:**
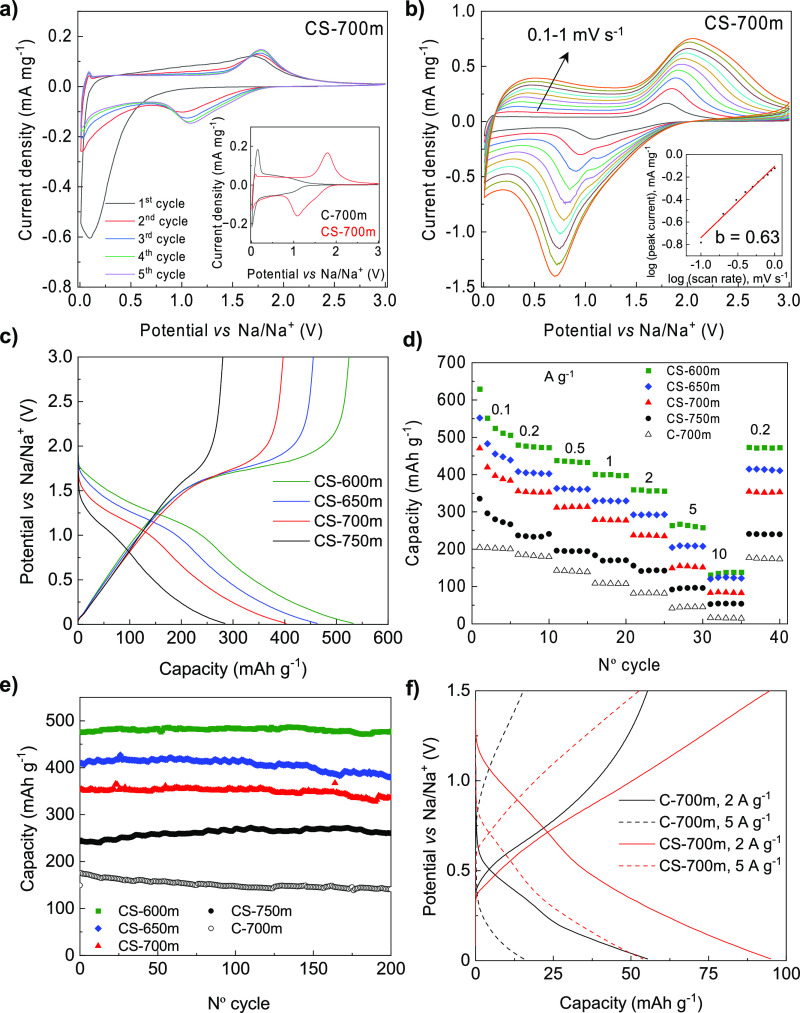
(a) Cyclic voltammograms at 0.1 mV s^–1^ for CS-700m (inset: comparison of the cyclic voltammogram with that of undoped C-700m), (b) cyclic voltammograms at different scan rates for CS-700m (inset: plot of *b*-value), (c) GCD profiles in the third cycle for the different S-doped sheets, (d) rate capability of the sheets, (e) long-term cycling stabilities for the non-doped (C-700m) and the S-doped carbon sheets at 0.2 A g^–1^, and (f) comparison of the 2nd GCD profiles acquired in the potential range of 1.5 to 0.01 V vs Na/Na^+^ at 2 and 5 A g^–1^ for C-700 and its S-doped counterpart CS-700.

The Na storage performance of the S-doped carbons was analyzed in half-cells using Na foil as a counter and reference electrode and as an electrolyte, the conventional solution of 1 M NaClO_4_ in EC/DEC (1:1 vol). Figure 3a shows the initial five CV curves at 0.1 mV s^–1^ for CS-700m (those for the rest of the S-doping temperatures can be found in Figure S4a–c). During the first sodiation process, there is an irreversible redox peak below ∼0.7 V vs Na/Na^+^, which disappears in the following cycles and is attributable to the electrolyte decomposition to form the SEI layer. From the second cycle and onward, a pair of redox peaks develop at around 1.1 V vs Na/Na^+^ during sodiation and ∼1.7–1.8 V vs Na/Na^+^ during de-sodiation. In addition, the intensity of those peaks decreases with the rise of the doping temperature and, consequently, the decrease of the sulfur content. Based on these considerations, as well as based on the fact that the cyclic voltammograms for the non-doped C-700m do not show any redox feature at such potentials (inset in Figure 3a), the pair of redox peaks can be assigned to the reactions between sodium and electrochemically active sulfur heteroatoms, which agrees with previous reports on S-doped carbons in Na-ion batteries^32,36,38^ and Na-ion capacitors.^14,17^ Specifically, that redox couple suggests that small sulfur species are the ones involved (e.g., Na_2_S_3_ and Na_2_S_4_) in the sodium storage process and no soluble long-chain polysulfides Na_2_S_*n*_ (5 < *n* < 6) are generated, which benefits the stability of the materials. This agrees with the fact that sulfur is covalently bonded in these materials (vide supra). The kinetics of the sulfur redox reactions was analyzed by using the power law dependence of the peak current (*i*) on scan rate (υ): *i*(*V*) = *k*·υ^*b*^, wherein *k* and *b* are adjustable parameters. The *b* value can be calculated by plotting log(*i*) vs log(υ). A value of *b* = 0.5 is indicative of a diffusion-controlled faradaic reaction, whereas *b* = 1 indicates a pseudocapacitive reaction.^39^ The value of *b* at the de-sodiation peak at ∼ 1.7–1.8 V vs Na/Na^+^ lies in the range of 0.57–0.70 (Figures 3b and S4d–f), indicating that sodium storage through redox reactions with sulfur heteroatoms has a mixed diffusion-capacitive control, with the diffusion control diminishing with the rise of temperature, which may be ascribed to the decreased contribution of the redox reactions and increase of the electronic conductivity (Table 1).

The GCD potential profiles are shown in Figure 3c. In accordance with the cyclic voltammograms, the curves corresponding to the S-doped carbon sheets display potential plateaus between 1.2 and 1.5 V vs Na/Na^+^ during discharge, whose length increases with the sulfur content. On the contrary, as shown in Figure S5, the undoped sheets exhibit the typical GCD profile of a hard carbon, with a plateau for potentials below 0.1 V vs Na/Na^+^ and a sloping region between 0.1 and 1 V vs Na/Na^+^. While the undoped sheets provide a reversible capacity (i.e., de-sodiation capacity) of only 200 mAh g^–1^ (148 mAh cm^–3^) at 0.1 A g^–1^ (ICE = 73%), its doped counterpart (CS-700m) doubles its capacity, providing 420 mAh g^–1^ (403 mAh cm^–3^, ICE = 69%) and the rest of the S-doped sheets between 300 and 550 mAh g^–1^ (285–522 mAh cm^–3^, ICE = 58–76). Noteworthily, the value of ICE of the S-doped sheets is above ∼70% (except for CS-750m) despite having surface areas of 430–480 m^2^ g^–1^, comparing very favorably with other doped or non-doped carbon materials with similar or lower values of surface area (see Table S1). Also important is that, at a high rate of 10 A g^–1^, the S-doped carbon sheets are still able to provide a reversible capacity in the ∼55–140 mAh g^–1^ range (53–133 mAh cm^–3^), compared to just ∼15 mAh g^–1^ for the undoped sheets (see Figure 3d), indicating a significant contribution of the sulfur redox reactions still at high rates. When the discharge current is set back to 0.2 A g^–1^ after cycling at high rates, the reversible capacity of the materials is completely restored to the initial level (240–470 mAh g^–1^), suggesting that the structure of the materials remains unaltered after fast sodiation and de-sodiation. A further proof of the stability of the S-doped sheets is provided by their long-term cycling at 0.2 A g^–1^ (Figure 3e), which shows a capacity retention above 92% after 200 cycles for all of them (81% for C-700m).

Figure 3c evidences that an important part of the reversible capacity of the S-doped materials is delivered at potentials above 1.5 V vs Na/Na^+^, especially in the case of samples with a high sulfur content that exhibit a long plateau above 1.5 V vs Na/Na^+^. However, the usual potential range of operation of the negative electrode in a NIC is from 1.5 to 0.01 V vs Na/Na^+^. A closer look at Figure 3c shows that the material showing the highest reversible capacity in that potential range is CS-700m, with its capacity also being above that of C-700m. Accordingly, this material would be the optimum one for assembling a NIC. In order to confirm these results, CS-700m and C-700m were cycled with a cut-off potential of 1.5 V vs Na/Na^+^. As can be seen in Figure S6, the reversible capacity of CS-700m is always above that of C-700m, and its rate capability is also much better. Figure 3f further highlights the superiority of CS-700m at high rates, providing lower de-sodiation potentials than C-700m. These observations ratify the beneficial effects of the S-doping treatment within the practical operation potential range and current rates of NIC negative electrodes, creating abundant sites for Na storage both above and below 1.5 V vs Na/Na^+^.

### 3.2 Structural, Chemical, and Electrochemical Properties of the Positive Electrode Material

**Figure 4:**
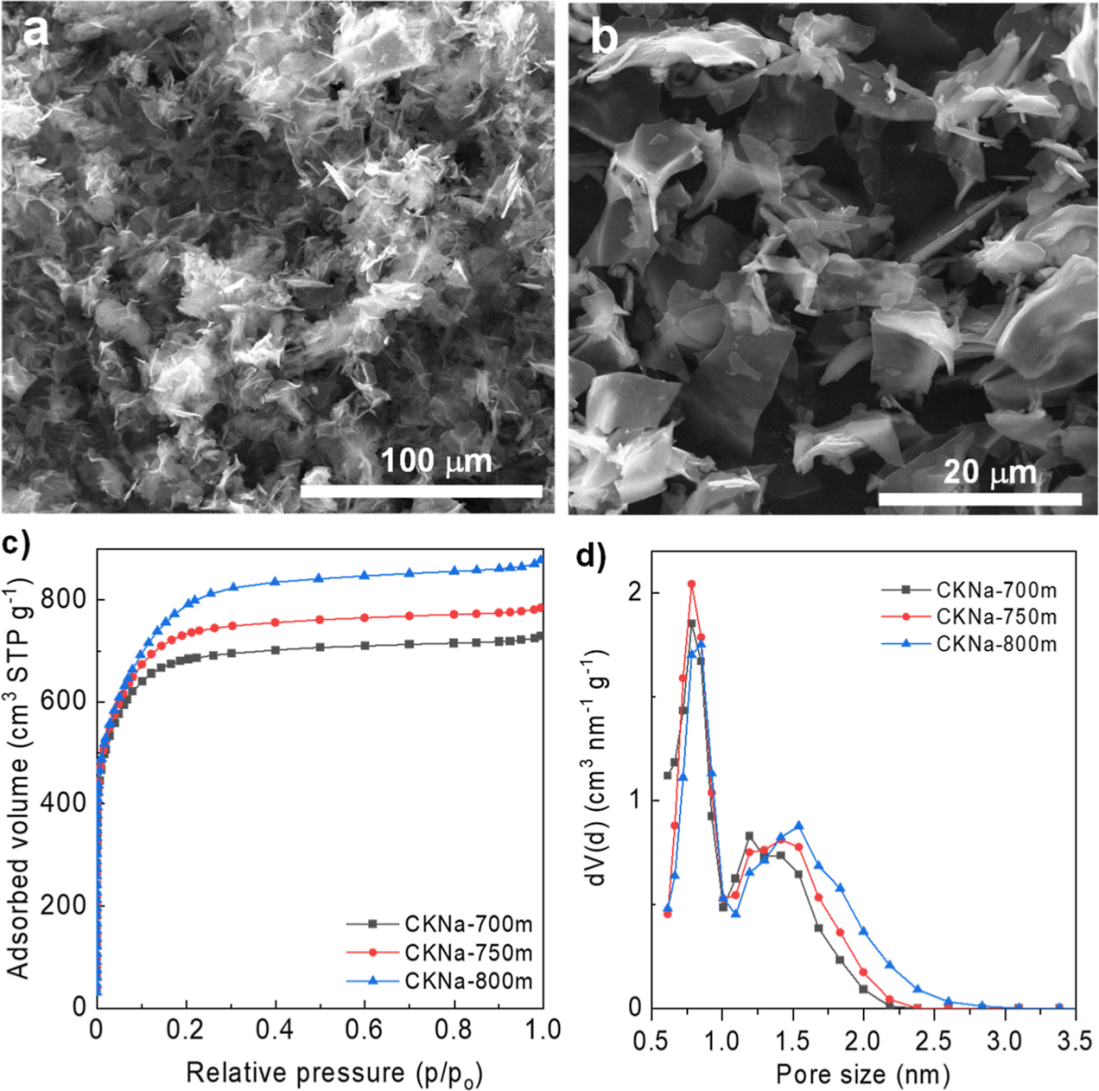
SEM micrographs of (a) CKNa-800 and (b) CKNa-800m and (c) N_2_ adsorption isotherms and (d) QSDFT PSD of the porous carbon sheets.

A two-step chemical activation approach was adopted to produce the highly porous cork-derived sheets that will serve as the positive electrode material. Alkaline hydroxides, especially potassium hydroxide, are well-known for their effectiveness as activating agents, generating highly porous materials with specific surface areas (*S*_BET_) above 2000 m^2^ g^–1^. However, they are very aggressive and do not allow us to retain the original morphology of the carbon precursor, leading normally to the same final morphology consisting of particles with conchoidal cavities.^40^ Thus, in order to stabilize the morphology of the cork, it was first pre-carbonized at 600 °C. It should be noted that this pre-carbonization step, besides stabilizing the structure, allows us to obtain a high yield in the activation step (>ca. 50%), which supposes an advantage from the environmental point of view given that, for a given production, a lower amount of toxic alkaline hydroxide is required. In this work, a mixture of KOH and NaOH has been used since, as will be shown later, it allows us to obtain a higher pore development than KOH. This fact may be explained by the higher reactivity/etching power of the mixture, as inferred from the lower activation yield (see Tables 2 and S2). In addition, NaOH is cheaper than KOH, which benefits the process from the economical point of view. As can be seen in Figure 4a, many sheets can already be seen in the material before the ball-milling step, which may be ascribed to the penetration of melted KOH/NaOH into the 3D structure of the pre-carbonized cork, causing already some partial de-construction. After a mild ball-milling step, further transformation into 2D sheets is achieved, as proved by Figure 4b, increasing the packing density by 10–15% (see Tables 2 and S2). It should be noted that this ball-milling step causes some decrease in porosity, but it is not significant (<5% by comparing data in Tables 2 and S2). Indeed, analysis through N_2_ physisorption proves the highly porous nature of these carbon sheets, which possess large BET surface areas in the ∼2600–2900 m^2^ g^–1^ range and pore volumes in the range of 1.2–1.3 cm^3^ g^–1^ (Table 2). On the contrary, the materials obtained by conventional KOH activation possess lower BET surface areas in the 2100–2700 m^2^ g^–1^ range and pore volumes below 1.2 cm^3^ g^–1^ (Table S2). Importantly, the activation yield is as high as 49–58% despite such high porosity developments (Table 2). Figure 4c reveals that the materials exhibit a type Ib isotherm, indicative of a microporous material with the presence of some small mesopores. This can be clearly seen in the PSDs in Figure 4d, which further show a progressive increase of the mesopore content with the increase of the activation temperature. Nevertheless, the materials are highly microporous (>87% of the pore volume corresponds to the volume of micropores), which leads to reasonable packing densities of around 0.5 cm^3^ g^–1^.

**Table 2:** Physico-Chemical Properties of the Highly Porous Carbon Sheets

carbon material	global synthesis yield (%)^a^	textural properties			packing density (g cm^–3^)^d^	electronic conductivity (S m^–1^)^d^
		*S*_BET_ (m^2^ g^–1^)	*V*_p_ (cm^3^ g^–1^)^b^	*V*_microp_ (cm^3^ g^–1^)^c^		
CKNa-700m	12 (58)	2590	1.17	1.07	0.54	100
CKNa-750m	11 (55)	2770	1.20	1.12	0.49	140
CKNa-800m	10 (49)	2910	1.34	1.17	0.46	190
CKNa-800mT		2750	1.24	1.09	0.45	250

a The yield corresponding to the activation step is indicated in parenthesis.

b Pore volume determined at (*P*/*P*_0_) = 0.95.

c Volume of pores < 2 nm determined from the QSDFT PSD.

d Determined at 7.1 MPa.

**Figure 5:**
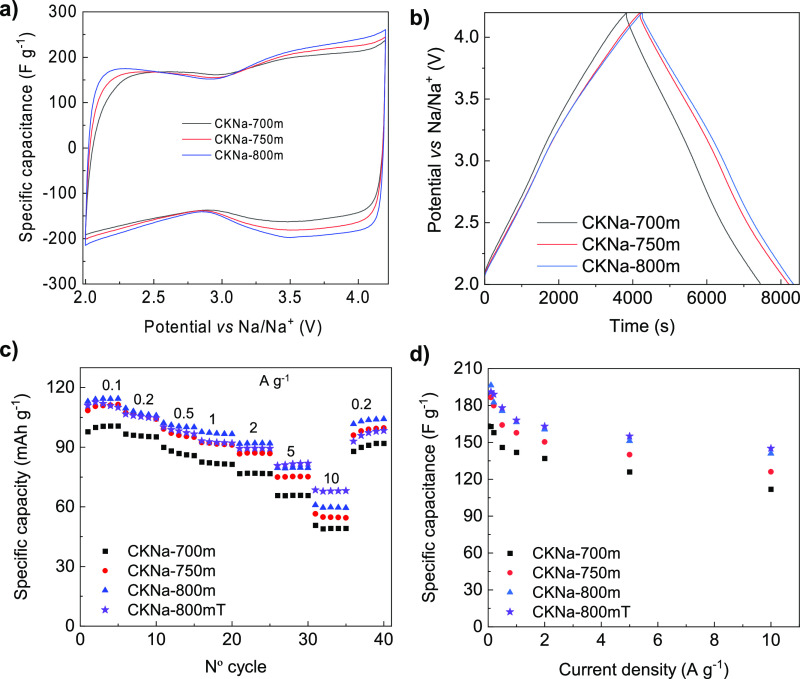
(a) Cyclic voltammograms at 2 mV s^–1^, (b) GCD cycles at 0.1 A g^–1^, (c) rate capability in terms of capacity, and (d) rate capability in terms of capacitance for the different porous carbon sheets.

The electrochemical performance of the carbon sheets was assessed in half-cells against Na metal in the potential range of 2–4.2 V vs Na/Na^+^. The cyclic voltammograms at 2 mV s^–1^ in Figure 5a exhibit a rectangular shape characteristic of the EDLC behavior, the shape being preserved for higher sweep rates (Figure S7). The narrowing at ca. 2.8 V vs Na/Na^+^ indicates the adsorption of ClO_4_^–^ anions at potentials higher than 2.8 vs Na/Na^+^, while Na^+^ cations are adsorbed below that potential. Large capacitances of around 200 F g^–1^ are obtained on account of the large surface area of the materials and their adequate micropore size distributions. The EDLC behavior is further confirmed by the symmetric triangular GCD cycles for low (Figure 5b) and intermediate-high discharge currents (Figure S8). The rate capability, as evaluated from the GCD cycles, is shown in Figure 5c,d in terms of capacity and capacitance, respectively. As can be seen, the materials show large specific capacity/capacitance values at 0.1 A g^–1^ in the range of 100–114 mAh g^–1^/163–196 F g^–1^ while keeping 50–60 mAh g^–1^/112–140 F g^–1^ at a high rate of 10 A g^–1^ thanks to the short ion diffusion distances in these sheets (<200 nm). Figure 5 reveals that the best-performing positive electrode material is the one synthesized at 800 °C that combines the largest amount of micropores, the highest proportion of mesopores, and the highest electronic conductivity (Table 2). In order to further improve the electrochemical performance of the positive electrode material in terms of capacity retention at high current rates, a post-synthesis thermal treatment at 800 °C was applied to the selected CKNa-800m material (sample labeled CKNa-800mT), as detailed in the Experimental Section. This second thermal treatment brought along a certain narrowing of the microporosity, with the consequent decrease in the BET-specific surface area (see Table 2 and Figure S9a,b). As a consequence, the treated sample registered a slightly lower capacity at the lowest charge and discharge rates (Figure 5c). On the upside, this thermal treatment caused an increase in the electronic conductivity (Table 2) and thereby an improvement in the charge transfer properties of the material (Figure S9c). Hence, it showed better capacity retention (ca. 70 mAh g^–1^ at 10 A g^–1^) at the high current densities at which hybrid capacitors are aimed to work.

### 3.3 Sodium-Ion Capacitor

**Figure 6:**
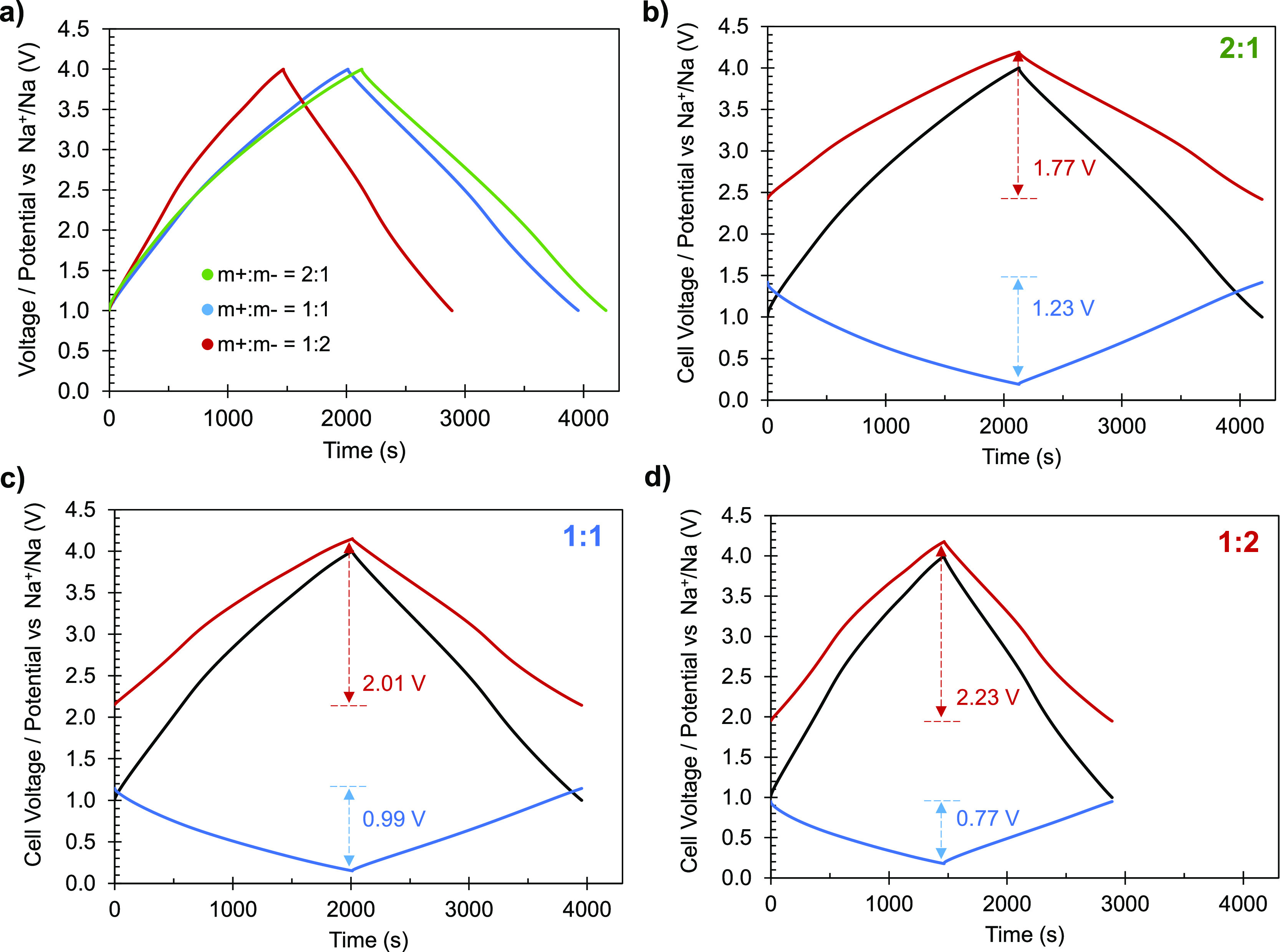
(a) Galvanostatic plots of the full cells charged and discharged at a current density of 0.1 A g^–1^ and (b–d) potential profiles of the negative (blue) and positive (red) electrodes at the same current density for the different NICs.

Based on the results discussed in previous sections, sodium-ion capacitors were assembled with CS-700m as the negative electrode material and CKNa-800mT for the positive electrode. Optimization of the energy-power characteristics of the full cell was assessed by the evaluation of different positive-to-negative electrode active mass ratios, namely, 2:1, 1:1, and 1:2, keeping constant the total active mass in the device. Prior to NIC assembling, both positive and negative electrodes were independently pre-cycled against sodium metal, as detailed in the Experimental Section, to minimize irreversible sodium losses. The full cell was first galvanostatically charged and discharged in the 1–4 V range at increasing current densities. Figure 6a shows the galvanostatic plots recorded at a low current density of 0.1 A g^–1^. In all cases, the voltage plot of the full cells is triangular, which confirms the essentially capacitive nature of the hybrid devices. At this current rate, the NIC with the highest positive-to-negative mass ratio (the 2:1 NIC) shows the longest charge and discharge times, indicative of a higher charge storage capacity. This can be assigned to a better capacity match between the lighter negative electrode—with a higher capacity—and the heavier positive electrode. Accordingly, a deeper utilization of the negative electrode was recorded in the 2:1 NIC, drawing a potential swing of 1.23 V, which decreased down to 0.99 and 0.77 V for the 1:1 and 1:2 electrode mass ratios, respectively (Figure 6b–d).

**Figure 7:**
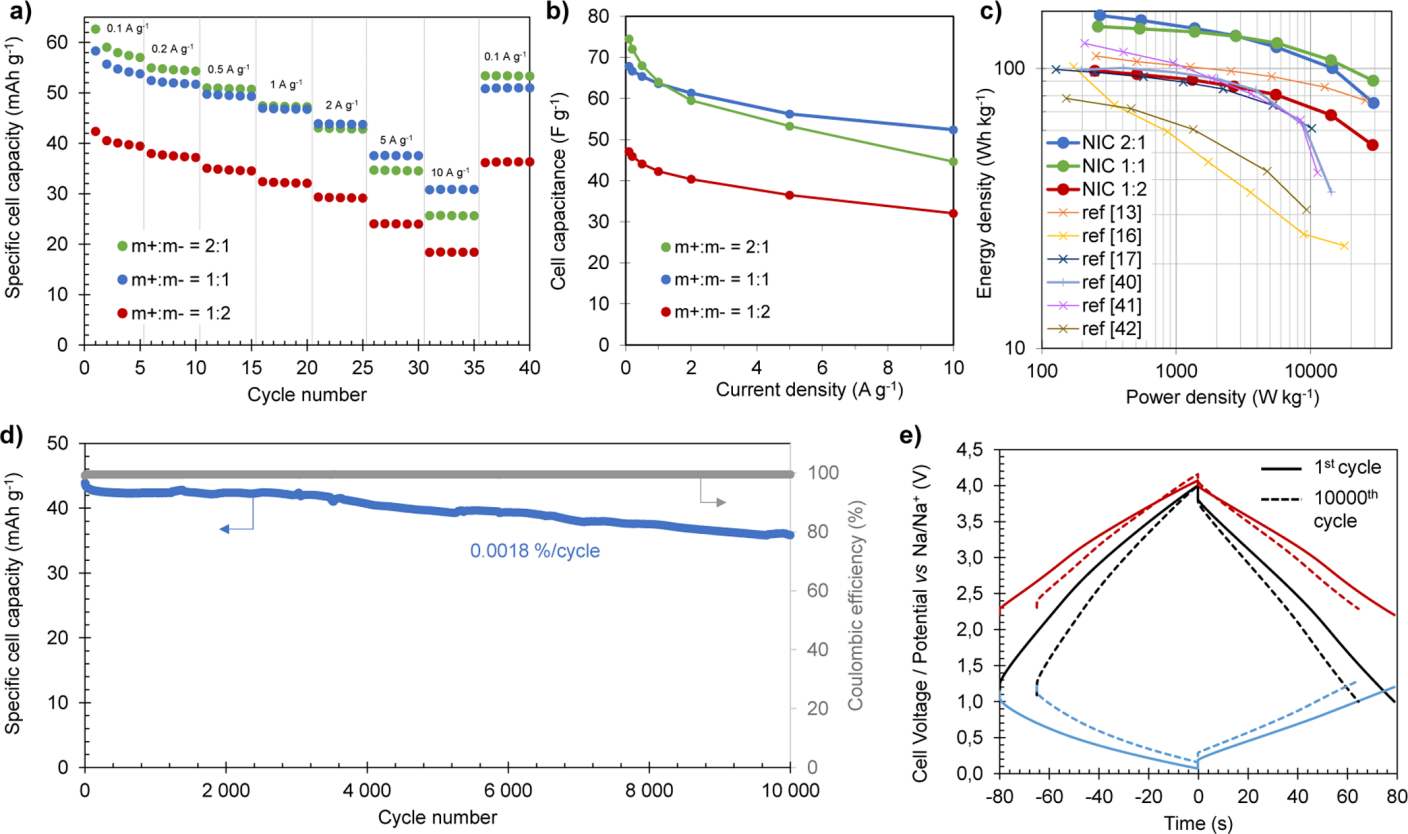
Rate capability in terms of (a) capacity and (b) capacitance, (c) Ragone plot of the NICs with different electrode mass ratios, (d) galvanostatic cycling stability test at 2 A g^–1^ for the 1:1 NIC, and (e) electrode potential profiles recorded at the first (solid) and 10,000th (dashed) cycle.

Figure 7a,b shows the evolution of the capacity and capacitance of the full cells at different current densities. The 2:1 and 1:1 NICs achieve high capacity (capacitance) values of 63 mAh g^–1^ (75 F g^–1^) and 58 mAh g^–1^ (68 F g^–1^), respectively, while the 1:2 NIC has a considerably lower capacity of 42 mAh g^–1^ (47 F g^–1^). This decrease in capacity for lower positive-to-negative electrode mass ratios than 1:1 has been confirmed over different intermediate mass ratios (Figure S10) and can be ascribed to the oversizing and underuse of the negative electrode. At increasing current densities, the 1:1 NIC is able to retain better the capacity, delivering as much as 31 mAh g^–1^ (52 F g^–1^) at a fast charge–discharge rate of 10 A g^–1^. Such a high capacity retention of 53% (77% in terms of capacitance) is the result of a better kinetic match between the positive and negative electrodes under fast discharge rates. Thus, the 1:1 ratio can be regarded as the optimum considering that hybrid capacitors are meant to operate under short charge and discharge times. A further decrease in the electrode mass ratio does not bring any improvement in terms of capacity retentions but lower capacities in the whole range of current densities (Figure S10).

The values of energy and power density achieved by the different NICs are collected in the Ragone plot shown in Figure 7c. At the lowest current rate, the 2:1 and 1:1 NICs deliver 155 and 141 Wh kg^–1^, respectively, and the cell with mass matching electrodes shows the best performance under the high-power regime, providing as much as 90 Wh kg^–1^ at a high-power density of 29 kW kg^–1^ (discharge time = 11 s). The 1:1 NIC not only possesses an excellent energy and power performance that compares favorably with other recently reported dual carbon Na-ion capacitors^14,17,18,41−43^ but also displays a very stable cyclability upon repetitive galvanostatic charge and discharge. As shown in Figure S11, the three full cells showed a stable cyclability. Particularly, the 1:1 NIC could be cycled for 10,000 cycles at a current density of 2 A g^–1^ experiencing a capacity fade of only 0.0018% per cycle, retaining 82% of its initial capacity at the end of the cycling test (Figure 7d). As depicted in Figure 7e, the electrode potential swings remain fairly steady during the whole stability test, showing only a slight upward shifting that guarantees the safe operation of the capacitor.

## 4 Conclusions

A green and simple procedure for the preparation of carbon sheets heavily doped with sulfur (S > 14 wt %) and with good sodium storage properties has been developed. It is based on the heat treatment at relatively low temperatures of 600–750 °C of pre-carbonized cork with sulfur as an earth-abundant, cost-effective, and environmentally benign S-dopant. A final mild ball-milling process is applied in order to produce 2D sheets from the 3D natural cellular structure of the cork. The thus-synthesized materials combine a highly disordered microstructure with enlarged interlayer spacing, a high sulfur content (14–27 wt %), short solid-state diffusion pathways arising from their 2D morphology, and a high packing density (∼1 g cm^–3^). As a result, these materials provide large Na storage capacities in the range of 300–550 mAh g^–1^ (285–522 mAh cm^–3^) at 0.1 A g^–1^ and still 55–140 mAh g^–1^ (53–133 mAh cm^–3^) at 10 A g^–1^.

A two-step chemical activation approach has been rationally designed to produce highly porous carbon sheets (*S*_BET_ > 2700 m^2^ g^–1^) from the same carbon precursor by using a mixture of sodium and potassium hydroxides. These materials showed a good capacitive performance, providing high ClO_4_^–1^ anion storage capacities/capacitances of up to 100–114 mAh g^–1^/163–196 F g^–1^ at 0.1 A g^–1^ and 50–60 mAh g^–1^/112–140 F g^–1^ at a high rate of 10 A g^–1^.

A NIC assembled with the optimized sheet carbons by using a positive-to-negative electrode mass ratio of 1 provided the best energy/power performance, storing 141 Wh kg^–1^ at low power and as much as 90 Wh kg^–1^ at a high power density of 29 kW kg^–1^ (discharge time = 11 s). These favorable energy/power characteristics were combined with robust cycling stability, experiencing a capacity fade of only 0.0018% per cycle for 10,000 cycles at a current density of 2 A g^–1^.

## Supporting Information Available

ICE values of negative electrode carbon materials used in SIBs and SICs; physico-chemical properties of the highly porous carbon nanosheets before the ball-milling step; SEM micrographs of pristine cork, cork carbonized at 400 °C, and materials doped at 600 and 750 °C (including also their EDX elemental mappings); XRD patterns corresponding to the non-doped and S-doped carbon nanosheets; deconvolution of the first-order Raman spectra; textural and electrochemical characterization for the positive electrode material before and after the thermal post-treatment; cyclic voltammograms at different sweep rates for S-doped and highly porous carbons; GCD profiles for the un-doped and porous carbon nanosheets; rate capability of CS-700m and C-700m in the potential range of 0.01 to 1.5 V vs Na/Na^+^; and rate capability and cycling stability of full cells with different positive-to-negative electrode mass ratios (PDF)
