# Supplementary material for: Cork-Derived Carbon Sheets for High-Performance Na-Ion Capacitors
Source: ACS Appl Energy Mater. 2023 Jul 17;6(15):8120–31. doi: 10.1021/acsaem.3c01212 (PMC10431350; doi:10.1021/acsaem.3c01212)
Supplement: Supplementary file 1 — ae3c01212_si_001.pdf [file ae3c01212_si_001.pdf]

Supporting Information

Cork-derived carbon sheets for high-performance Na-ion capacitors

María Dolores Casal, Noel Díez, Sara Payá, Marta Sevilla*

Instituto de Ciencia y Tecnología del Carbono (INCAR), CSIC, Francisco Pintado Fe 26, 33011 Oviedo, Spain

* Corresponding author. Tel: 34 985119002. E-mail: martasev@incar.csic.es

Table S1. Specific surface area and initial coulombic efficiency (ICE) for carbon negative electrode materials used in sodium-ion batteries or sodium-ion capacitors.

Material	Surface area (m^2 g^{-1})	ICE (%)	Ref.
CS-600m	430	76	This work
CS-650m	454	72	This work
CS-700m	484	69	This work
CS-750m	534	58	This work
N-doped carbon/carbon nanocomposite	415	45	[1]
N/S co-doped hollow carbon nanofiber	400	35	[2]
S-doped graphene hollow spheres	320	36	[3]
N-doped hollow carbon spheres	317	50	[4]
N-doped porous carbon	129	67	[5]
N-doped 3D porous carbon	517	29	[6]
Garlic-derived hard carbon	196	35	[7]
Heavily S-doped carbon	526	68	[8]
Heavily S-doped disordered carbon	117	63	[9]
Heavily S-doped carbon nanosheets	231	58	[10]

References:

(1) Yan, R.; Leus, K.; Hofmann, J. P.; Antonietti, M.; Oschatz, M. Porous Nitrogen-Doped Carbon/Carbon Nanocomposite Electrodes Enable Sodium Ion Capacitors with High Capacity and Rate Capability. *Nano Energy* **2020**, *67*, 104240. https://doi.org/https://doi.org/10.1016/j.nanoen.2019.104240.

(2) Liao, K.; Wang, H.; Wang, L.; Xu, D.; Wu, M.; Wang, R.; He, B.; Gong, Y.; Hu, X. A High-Energy Sodium-Ion Capacitor Enabled by a Nitrogen/Sulfur Co-Doped Hollow Carbon Nanofiber Anode and an Activated Carbon Cathode. *Nanoscale Adv.* **2019**, *1* (2), 746–756. https://doi.org/10.1039/C8NA00219C.

(3) Thangavel, R.; Kannan, A. G.; Ponraj, R.; Yoon, G.; Aravindan, V.; Kim, D.-W.; Kang, K.; Yoon, W.-S.; Lee, Y.-S. Surface Enriched Graphene Hollow Spheres towards Building Ultra-High Power Sodium-Ion Capacitor with Long Durability. *Energy Storage Mater.* **2020**, *25*, 702–713. https://doi.org/https://doi.org/10.1016/j.ensm.2019.09.016.

(4) Liu, L.; Sun, X.; Dong, Y.; Wang, D.; Wang, Z.; Jiang, Z.; Li, A.; Chen, X.; Song, H. N-Doped Hierarchical Porous Hollow Carbon Spheres with Multi-Cavities for High Performance Na-Ion Storage. *J. Power Sources* **2021**, *506*, 230170. https://doi.org/https://doi.org/10.1016/j.jpowsour.2021.230170.

(5) Wang, Z.; Wang, X.; Bai, Y.; Yang, H.; Li, Y.; Guo, S.; Chen, G.; Li, Y.; Xu, H.; Wu, C. Developing an Interpenetrated Porous and Ultrasuperior Hard-Carbon Anode via a Promising Molten-Salt Evaporation Method. *ACS Appl. Mater. Interfaces* **2020**, *12* (2), 2481–2489. https://doi.org/10.1021/acsami.9b18495.

(6) Zhang, L.; Sun, J.; Zhao, H.; Sun, Y.; Dai, L.; Yao, F.; Fu, Y.; Zhu, J. Gas

Expansion-Assisted Preparation of 3D Porous Carbon Nanosheet for High-Performance Sodium Ion Hybrid Capacitor. *J. Power Sources* **2020**, *475*, 228679. https://doi.org/https://doi.org/10.1016/j.jpowsour.2020.228679.
(7) Liu, H.; Liu, X.; Wang, H.; Zheng, Y.; Zhang, H.; Shi, J.; Liu, W.; Huang, M.; Kan, J.; Zhao, X.; Li, D. High-Performance Sodium-Ion Capacitor Constructed by Well-Matched Dual-Carbon Electrodes from a Single Biomass. *ACS Sustain. Chem. Eng.* **2019**, *7* (14), 12188–12199. https://doi.org/10.1021/acssuschemeng.9b01370.
(8) Wu, T.; Jing, M.; Yang, L.; Zou, G.; Hou, H.; Zhang, Y.; Zhang, Y.; Cao, X.; Ji, X. Controllable Chain-Length for Covalent Sulfur–Carbon Materials Enabling Stable and High-Capacity Sodium Storage. *Adv. Energy Mater.* **2019**, *9* (9), 1803478. https://doi.org/https://doi.org/10.1002/aenm.201803478.
(9) Li, W.; Zhou, M.; Li, H.; Wang, K.; Cheng, S.; Jiang, K. A High Performance Sulfur-Doped Disordered Carbon Anode for Sodium Ion Batteries. *Energy Environ. Sci.* **2015**, *8* (10), 2916–2921. https://doi.org/10.1039/c5ee01985k.
(10) Zhao, G.; Yu, D.; Zhang, H.; Sun, F.; Li, J.; Zhu, L.; Sun, L.; Yu, M.; Besenbacher, F.; Sun, Y. Sulphur-Doped Carbon Nanosheets Derived from Biomass as High-Performance Anode Materials for Sodium-Ion Batteries. *Nano Energy* **2020**, *67*, 104219. https://doi.org/https://doi.org/10.1016/j.nanoen.2019.104219.

Table S2. Physico-chemical properties of the porous carbons produced by KOH activation and the highly porous carbons produced by KOH/NaOH activation before the ball-milling step.

Carbon material	Yield (%) [a]	Textural properties		Packing density [c] (g cm^{-3})
		S_{BET} (m^2 g^{-1})	V_p [b] (cm^3 g^{-1})	
CK-700m	14 (69)	2080	0.84	-
CK-800m	13 (61)	2720	1.20	-
CKNa-700	12 (58)	2730	1.17	0.48
CKNa-750	11 (55)	2800	1.22	0.41
CKNa-800	10 (49)	2950	1.36	0.40

[a] The yield corresponding to the activation step is indicated in parenthesis. [b] Pore volume determined at (P/P_0) = 0.95. [c] Determined at 7.1 MPa

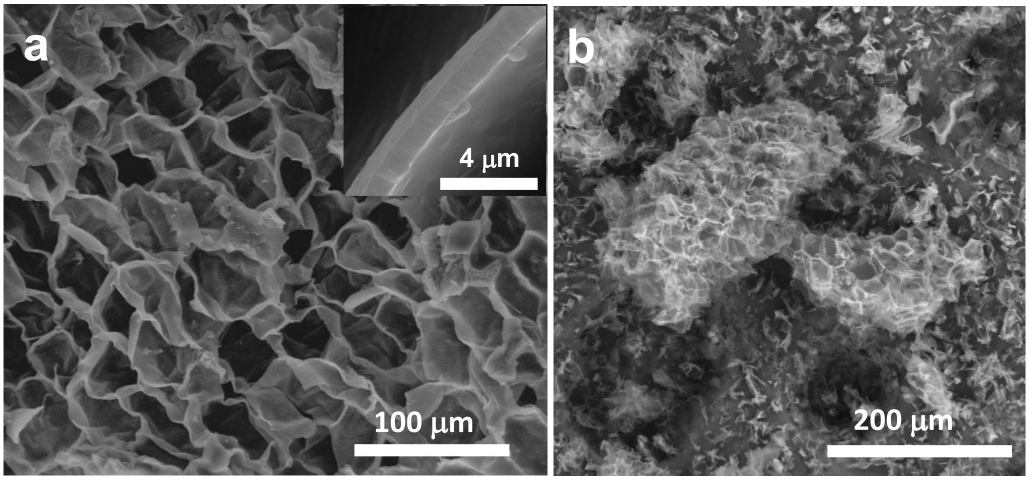

Figure S1. SEM pictures of a) pristine cork and b) cork carbonized at 400 ºC.

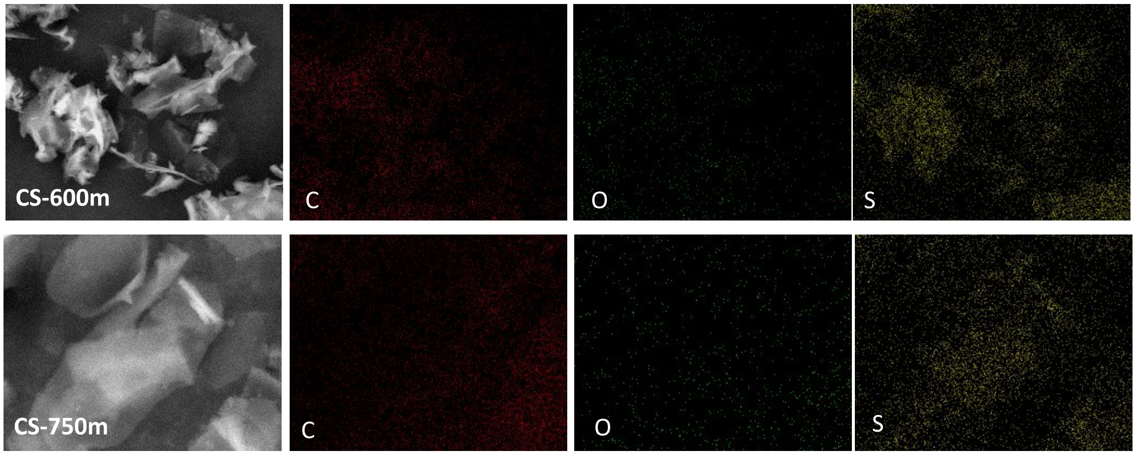

Figure S2. SEM micrographs corresponding to CS-600m and CS-750m with their corresponding EDX mappings for carbon, oxygen and sulfur.

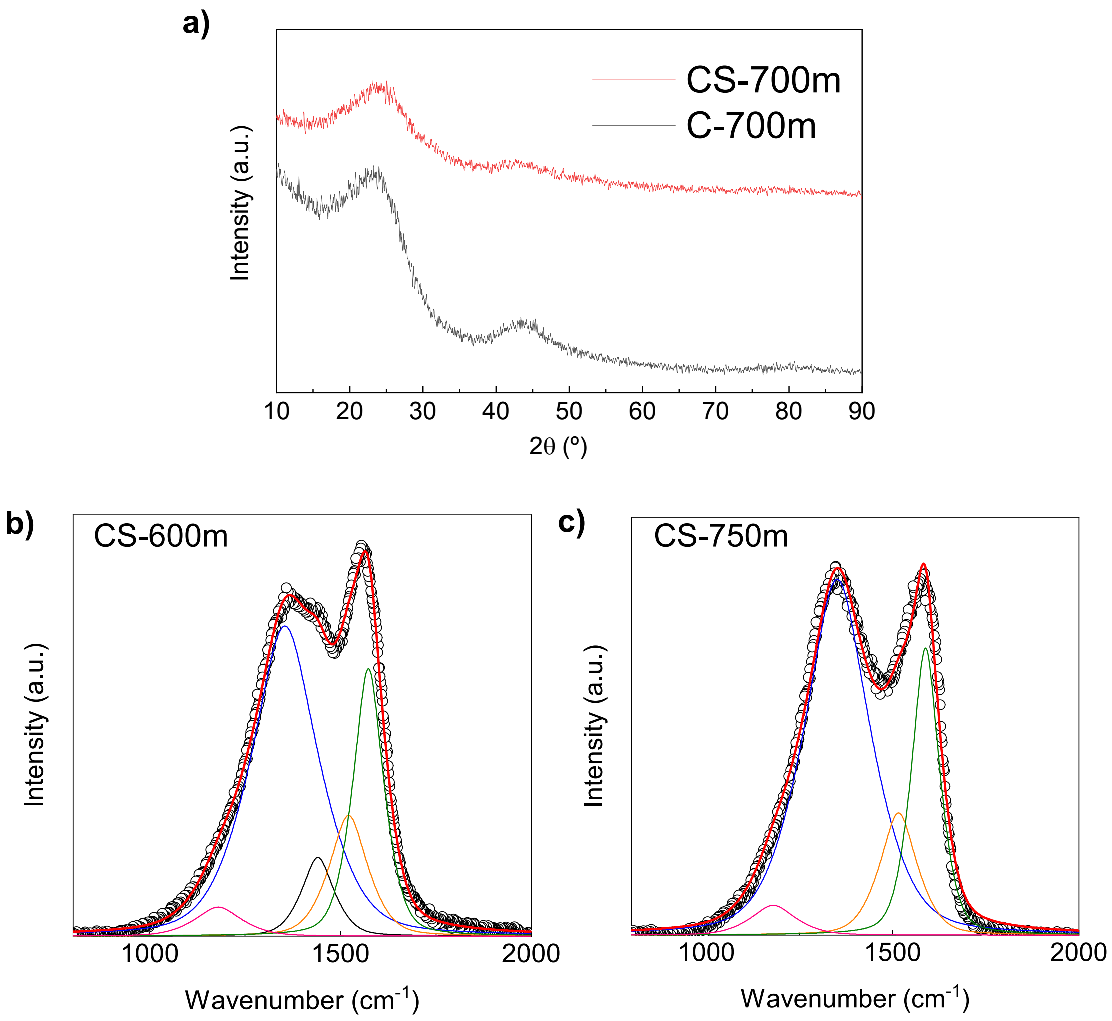

Figure S3. a) Comparison of the XRD patterns corresponding to the non-doped (C-700m) and S-doped carbon nanosheets, b) deconvolution of the first-order Raman spectrum of CS-600m and c) deconvolution of the first-order Raman spectrum of CS-750m.

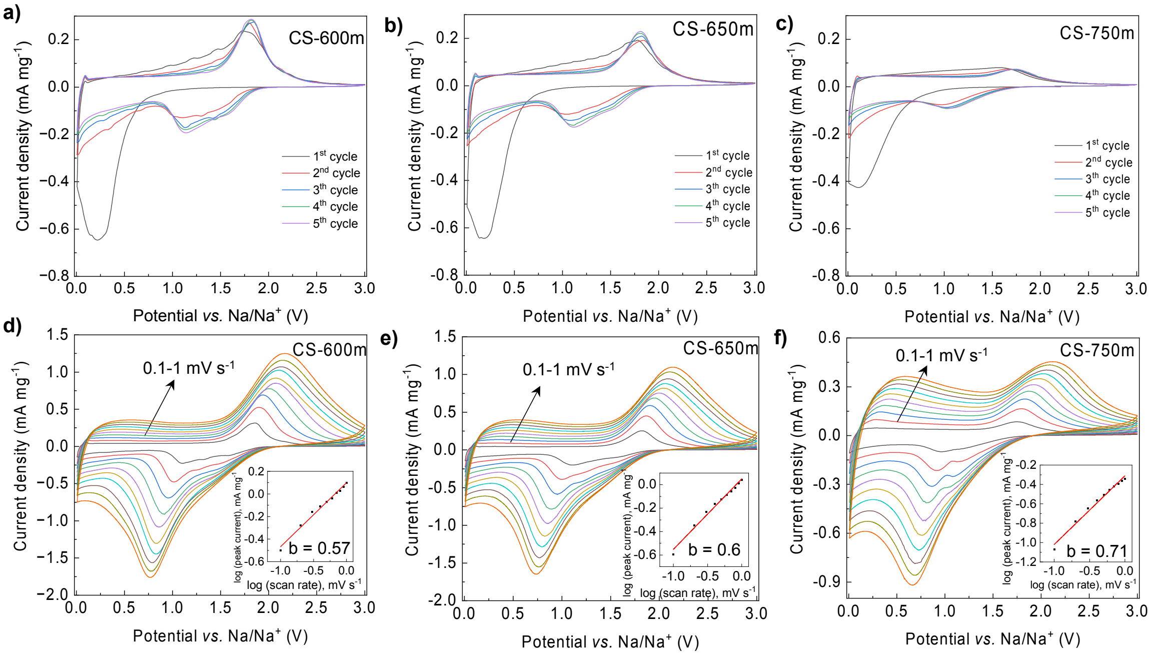

Figure S4. Cyclic voltammograms at 0.1 mV s^{-1} for a) CS-600m, b) CS-650m and c) CS-750m, and cyclic voltammograms at different sweep rates for d) CS-600m, e) CS-650m and f) CS-750m.

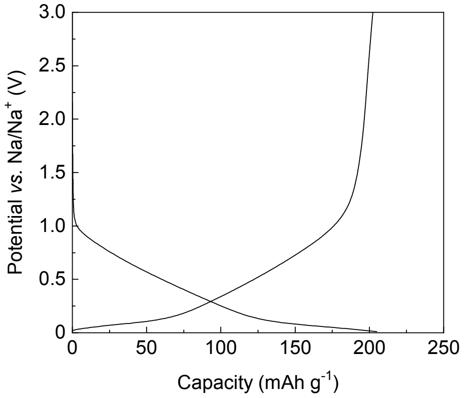

Figure S5. GCD profile in the third cycle for the un-doped carbon sheets C-700m.

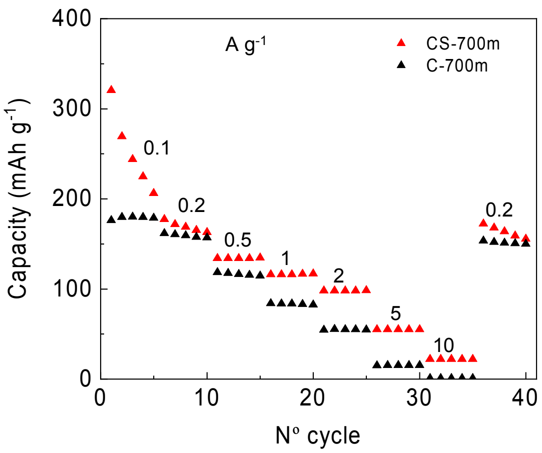

Figure S6. Rate capability of the CS-700m and C-700m carbon sheets in the potential range of 0.01 to 1.5 V *vs.* Na/Na^+.

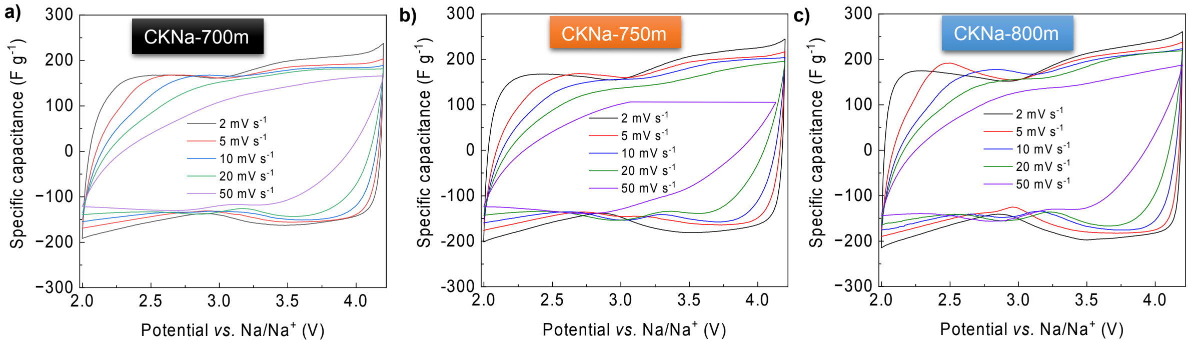

Figure S7. CVs at different sweep rates for a) CKNa-700m, b) CKNa-750m and c) CKNa-800m.

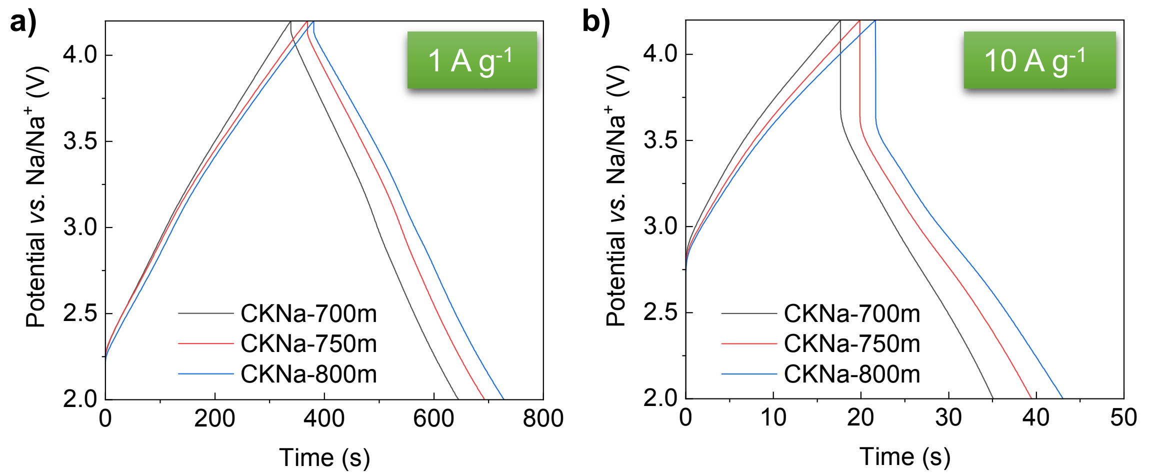

Figure S8. GCD profiles at a) 1 A g^{-1} and b) 10 A g^{-1} for the porous carbon nanosheets synthesized at different activation temperatures

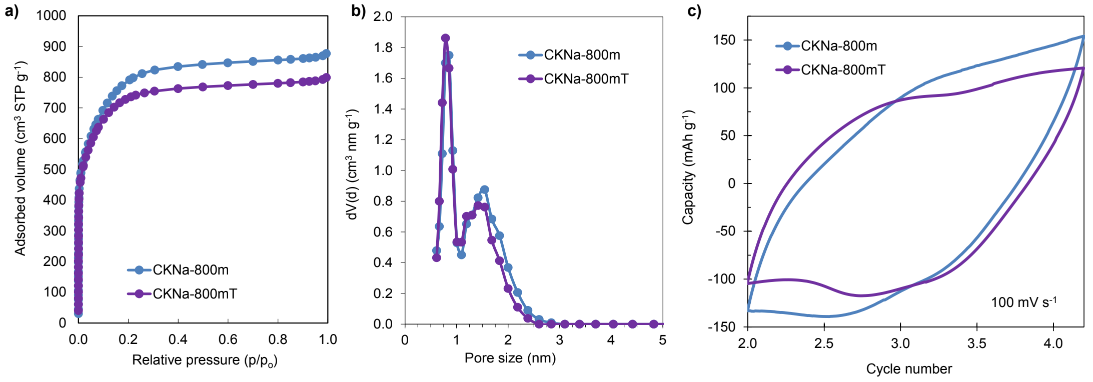

Figure S9. a) nitrogen isotherms and b) pore size distributions for the positive electrode material CKNa-800m before and after the thermal post-treatment at 800 ºC. c) Cyclic voltammetry experiments recorded for the positive electrode material CKNa-800m before and after the thermal post-treatment at a voltage sweep rate of 100 mV s^{-1}.

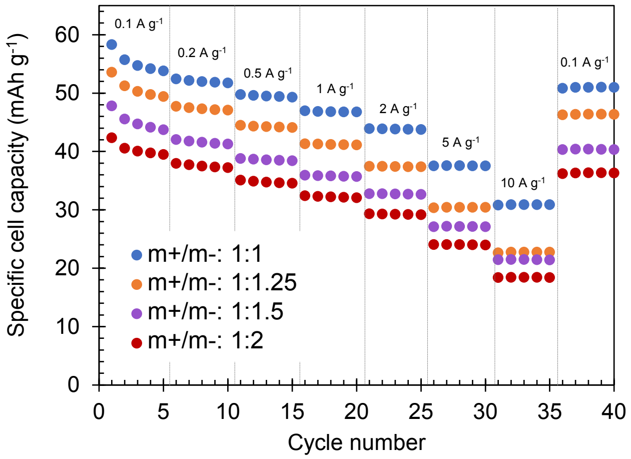

Figure S10. Evolution of capacity with the current density for full cells with different positive-to-negative electrode mass ratios of 1:1, 1:1.25, 1:1.5 and 1:2.

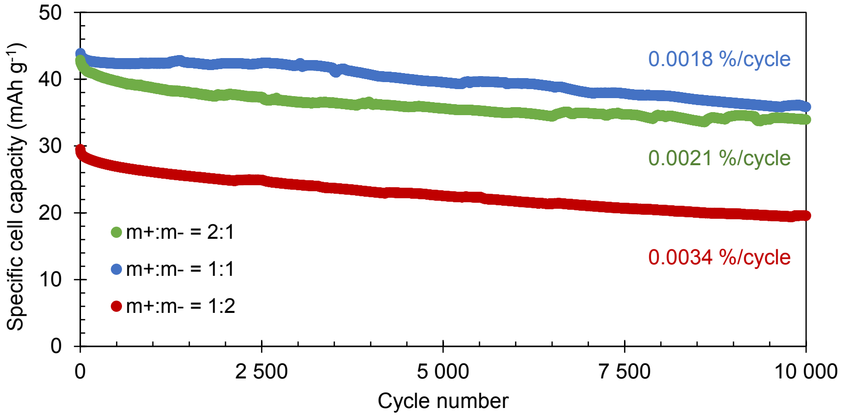

Figure S11. Galvanostatic cycling stability test for the different NICs (current density: 2 A g^{-1}) indicating the capacity fade in each case.
